# Synthesis of Controllable Superparamagnetic Nano Fe_3_O_4_ Based on Reduction Method for Colloidal Clusters of Magnetically Responsive Photonic Crystals

**DOI:** 10.3390/nano14100852

**Published:** 2024-05-14

**Authors:** Jun Chen, Mengdong Tu, Mengying Xu, Depeng Gong, Xi Li, Chaocan Zhang

**Affiliations:** 1 School of Materials Science and Engineering, Wuhan University of Technology, Wuhan 430070, China; 317093@whut.edu.cn (J.C.); 303549@whut.edu.cn (M.T.); whut165890@163.com (M.X.); gdp@whut.edu.cn (D.G.); 2 School of Chemistry, Chemical Engineering and Life Science, Wuhan University of Technology, Wuhan 430070, China

**Keywords:** magnetically responsive photonic crystals, reduction method, controllable, modification

## Abstract

In this paper, we designed and investigated a reduction-based method to synthesize controllably monodisperse superparamagnetic nano Fe_3_O_4_ colloidal clusters for magnetically responsive photonic crystals. It was shown that the addition of ascorbic acid (VC) to the system could synthesize monodisperse superparamagnetic nano Fe_3_O_4_ and avoided the generation of γ-Fe_2_O_3_ impurities, while the particle size and saturation magnetization intensity of nano Fe_3_O_4_ gradually decreased with the increase of VC dosage. Nano Fe_3_O_4_ could be rapidly assembled into photonic crystal dot matrix structures under a magnetic field, demonstrating tunability to various diffraction wavelengths. The nano Fe_3_O_4_ modified by polyvinylpyrrolidone (PVP) and silicon coated could be stably dispersed in a variety of organic solvents and thus diffracted different wavelengths under a magnetic field. This is expected to be applied in various scenarios in the field of optical color development.

## 1. Introduction

Photonic crystals (PCs) refer to a special optical material formed by the periodic arrangement of substances with different dielectric constants [1,2]. When PCs form a specific periodic dot structure by themselves or by changing external conditions, they are able to diffract a specific reflected wavelength, thereby controlling the propagation of light and modulating its properties [3]. Magnetic responsive photonic crystals (MRPCs) have become a new type of photonic manipulation due to the virtue of their instantaneous response, flexibility, adjustability, and ease of preparation [4]. Generally, monodisperse superparamagnetic nano Fe_3_O_4_ colloids are used as building blocks, which can be rapidly assembled into periodic lattice dot-array structures under the action of a magnetic field. By adjusting the magnetic field strength or the particle size of nano Fe_3_O_4_, flexible tuning of diffraction wavelengths can be achieved. On the other hand, superparamagnetic nano Fe_3_O_4_ in biomedicine, with its stable material property and good biocompatibility, can serve as magnetic fluid assisted hyperthermia and is expected to become an effective treatment method for cancer [5,6]. Therefore, this monodisperse superparamagnetic nanoparticle is widely used in the fields of color display [7,8], anti-counterfeiting [9,10], sensors [11,12], and biomedicine [13,14].

The traditional methods for the synthesis of Fe_3_O_4_ nanoparticles include the coprecipitation method [15,16,17], microemulsion method [18,19,20], and sol-gel method [21,22,23], etc. However, the Fe_3_O_4_ nanoparticles prepared by these three methods are usually uncontrollable, and the monodispersity cannot meet the requirements for the assembly of MRPCs; however, the solvothermal method is commonly used for the synthesis of monodisperse nano Fe_3_O_4_ to construct MRPCs due to its flexibility and adjustability. Li [24] successfully synthesized monodisperse nano Fe_3_O_4_ for the first time using the solvothermal method by utilizing ferric trichloride hexahydrate as the iron source, ethylene glycol and polyethylene glycol as the solvents and surfactants; the synthesized nano Fe_3_O_4_ exhibits superparamagnetic properties at room temperature, without remanent magnetism and coercivity. Yin’s research group [25,26,27] synthesized superparamagnetic nano Fe_3_O_4_ colloidal clusters using anhydrous ferric chloride as the iron source, diethylene glycol as the solvent, and polyacrylic acid as the surfactant. These clusters can be instantly assembled into photonic crystal structures under a magnetic field. Pan [28] synthesized monodisperse superparamagnetic Fe_3_O_4_ colloidal clusters using anhydrous ferric chloride as the iron source, ethylene glycol as the solvent, and Poly (4-styrene sulfonic acid copolymer maleic acid) sodium salt (PSSMA) as the surfactant, which can be instantaneously assembled into photonic crystals under a magnetic field. Zhang [29] synthesized monodisperse Fe_3_O_4_ nanoclusters using a mixture of EG and DEG (1:3) as the solvent, and sodium citrate as the surfactant, which exhibit superparamagnetism at room temperature and are able to diffract different ranges of wavelengths in the presence of a magnetic field. MRPCs based on spatial repulsion were prepared for the first time by Guan’s group [30,31]. In ethylene glycol, PVP was used as a surfactant to cover the surface of Fe_3_O_4_ nanoparticles, and tannic acid binding was used to further increase the stability at a later stage. The synthesized nanoparticles have good monodispersity and exhibit superparamagnetism at room temperature, and can be stably dispersed in a variety of solvents.

However, using the solvothermal method and similar raw materials as the system, some studies in the literature reported that the synthesized Fe_3_O_4_ nanoparticles have different results in monodispersity and superparamagnetism compared to the aforementioned literature [32,33,34,35,36,37,38,39,40]. Li [41] used anhydrous ferric chloride as the iron source, ethylene glycol as the solvent, and sodium citrate as the surfactant, and the particle size was adjusted by varying the amount of sodium citrate. Although the particle size decreases with the increase of sodium citrate, the synthesized Fe_3_O_4_ nanoparticles have a wide particle size distribution and exhibit soft magnetism at room temperature, with coercivity of 52 Oe, 51 Oe, and 35 Oe, respectively. Liu [42] also used polyacrylic acid as the surfactant and regulated the size of the product through the amount of water. However, the nanoparticles synthesized under various conditions show soft magnetic properties at room temperature with significant remanent magnetism and coercivity (minimum of 110 Oe). Moreover, the products synthesized under certain conditions have wide particle size distribution and poor monodispersity, which prevent them from assembling into photonic crystals. The nano Fe_3_O_4_ synthesized by Priyanka Saha [43] using PVP as a surfactant exhibits soft magnetism at room temperature, with a remanent magnetism of 20 emu/g and coercivity of 100 Oe, respectively. Liang [40] synthesized nano Fe_3_O_4_ using a mixture of ethylene glycol and diethylene glycol as solvents and polyethylene glycol as a surfactant, with a wide particle size distribution and exhibiting soft magnetism at room temperature, with the residual magnetism and coercivity being 13 emu/g and 89 Oe, respectively.

Our research group found that Fe^2+^ in the precursor is an essential component in the preparation of superparamagnetic nano Fe_3_O_4_ with photonic crystal effect and monodispersity by the solvothermal method using FeCl_3_ as the iron source, and proposed the nucleation theory of binuclear iron complexes [44]. In the process of preparing Fe_3_O_4_ MRPCs, a trace amount of Fe^2+^, which usually is present in varying content in the raw material FeCl_3_, will lead to the synthesized Fe_3_O_4_ nanoparticles differing and being difficult to control in terms of particle size, monodispersity, and magnetic properties. Therefore, this article proposes a method based on reduction to synthesize controllable monodisperse superparamagnetic nano Fe_3_O_4_ colloidal clusters for MRPCs; that is, ascorbic acid (VC) is used as a reductant to control the Fe^2+^ content in the system to synthesize superparamagnetic nano Fe_3_O_4_ with controllable particle size and polydispersity. The effects of different amounts of VC on the particle size, monodispersity, magnetic properties, and tunable optical properties of the synthesized nano Fe_3_O_4_ colloidal clusters were studied. Finally, the synthesized nano Fe_3_O_4_ particles were modified by PVP coated silica to make them stably dispersed in a variety of non-aqueous organic solvents.

## 2. Experimental Methods

### 2.1. Materials

Anhydrous ferric chloride (FeCl_3_, ≥99.0%), ethylene glycol (EG), anhydrous ethanol (EtOH), sodium hydroxide (NaOH), hydrogen peroxide (H_2_O_2_), ammonia (NH_3_·H_2_O), diethylene glycol (DEG), anhydrous methanol (MeOH), isopropyl alcohol (IPA), N-methyl pyrrolidone (NMP), N,N-dimethylformamide (DMF), and dimethyl sulfoxide (DMSO) were purchased from Shanghai Sinopharm Chemical Reagent Co. Anhydrous sodium acetate (NaAc), poly(4-styrenesulfonic acid maleic anhydride copolymer) sodium salt (PSSMA, MW ≈ 20,000, SS:MA = 1:1), anhydrous ferrous chloride (FeCl_2_ ≥ 99.5%), ascorbic acid (VC), tetraethyl orthosilicate (TEOS), and polyvinylpyrrolidone (PVP, K-30) were purchased from Shanghai Aladdin Biochemical Technology Co., Shanghai, China. Deionized water was self-made in the laboratory.

### 2.2. Preparation of Nano Fe_3_O_4_

In the modified solvothermal synthesis, 1.3 g of FeCl_3_, 2 g of PSSMA, and 6 g of NaAc were added to 80 mL of EG and heated while being stirred until complete dissolution, then diluted H_2_O_2_ was added to remove residual Fe^2+^ from the system. Next, the system was kept under nitrogen for half an hour, after which a certain amount of VC and 1.2 g of NaOH were added with stirring until complete dissolution to obtain the hot precursor. Finally, the precursor was transferred to a flask and heated in an oven at 190 °C for 10 h. After cooling to room temperature, the black particles were collected by a magnet and washed several times with alcohol and water, and eventually dispersed in deionized water.

### 2.3. Modification of Nano Fe_3_O_4_

PVP was added to co-modify in the conventional silicon coated process. An amount of 20 mL of Fe_3_O_4_ aqueous dispersion and 0.2 g of PVP was added to 110 mL of EtOH and stirred evenly in a flask. Then, 0.6 mL of TEOS was diluted with 25 mL of EtOH and transferred to a constant pressure funnel, adjusted to the appropriate speed to slowly drip into the flask while the mechanical stirring speed was kept at 1500 r/min. After the liquid in the funnel had finished dripping, the black particles were collected with a magnet and washed three times with EtOH before being dispersed as a whole in EtOH. The above process was repeated without adding PVP for modification in the system as a comparison.

### 2.4. Material Characterization

All digital photographs in this paper were taken by iPhone 12 and maintained at a certain lighting intensity. The structure of the precursors was analyzed using a UV–Vis spectrometer model Lamdba, Perkin Elmer, Waltham, MA, USA. The groups of products were analyzed using an infrared spectrometer (400–4000 cm^−1^) of Nicolet Nexus model of Thermo Fisher Scientific, Waltham, MA, USA. The particle size and potential of the products were measured using a Zetasizer Ultra model dynamic light dispersometer from Malvern Instruments Ltd., UK. The surface morphology of the synthesized and modified products was observed using a Tecnai G2 F20 type field emission transmission electron microscope from FEI, Hillsboro, OR, USA. The crystal shape of the products was tested using a D8 Advance type X-ray diffractometer from Brucker Technology GmbH, Saarbrucken, Germany. The chemical compositions of the synthesized products were analyzed using an X-ray photoelectron spectrometer model ESCALAB250Xi from Thermo Fisher Scientific, USA. The magnetic properties of the synthesized products were tested at room temperature using a vibrating sample magnetometer model 7404 from LakeShore, LA, USA. The reflectance spectra of the MRPCs were collected using a fiber optic spectrometer model ATP2000P from OPTISCO, Xiamen, China.

## 3. Results and Discussions

### 3.1. Preparation of Fe_3_O_4_ MRPCs Based on Reduction Method

**Figure 1:**
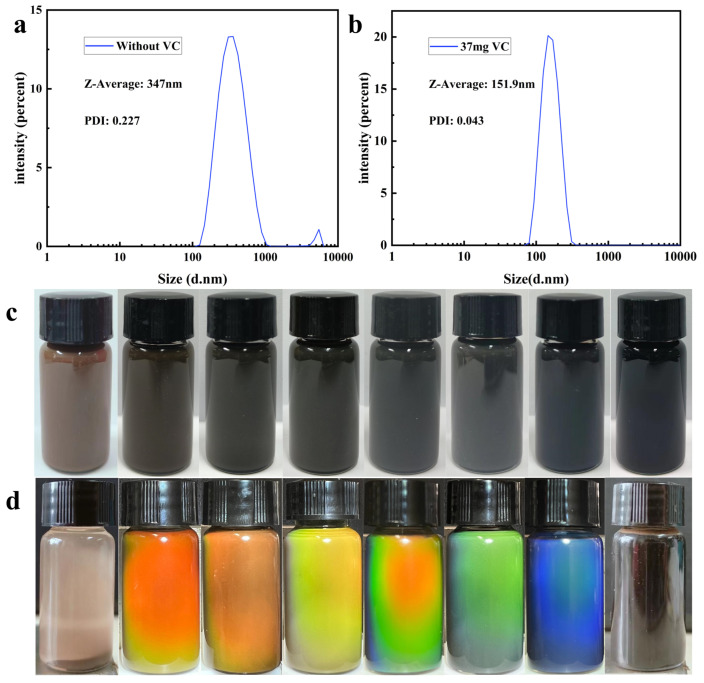
(**a**) Particle size distribution of products without VC in precursor; (**b**) particle size distribution of products with VC (37 mg) in precursor; (**c**) water dispersion photos of synthesized products with varying VC (increasing from left to right from 0) addition amounts; (**d**) optical photos of synthesized products with varying VC (increasing from left to right from 0) addition amounts under magnetic field.

In the process of preparing Fe_3_O_4_ MRPCs by the solvothermal method, Fe^2+^ in the precursor is an indispensable component and varying amounts of Fe^2+^ have an effect on the performance of Fe_3_O_4_ [44]. Therefore, we used VC as a reducing agent to reduce part of the Fe^3+^ in the system to Fe^2+^ and synthesized batches of different products by controlling the ratio of Fe^3+^ to Fe^2+^ by changing the content of VC, the properties of which are shown in Table 1. The Fe^2+^ in the raw material was first removed by hydrogen peroxide, and different products were obtained by adding varying amounts of VC. When no VC was added to the system (Sample No. 1), the average particle size of the synthesized product was large at 347 nm, with poor monodispersity and micron-sized agglomerates (Figure 1a). The product appeared brown in color (Figure 1c) and settled rapidly under a magnetic field without magnetic response to a photonic crystal diffraction effect (Figure 1d). When a certain amount of VC was added to the system, the synthesized product exhibited good monodispersity with uniform distribution as shown in Figure 1b. As the amount of VC was increased from 22 mg to 47 mg, the particle size of the products gradually decreased from 223.4 mm to 122.2 mm with remaining good monodispersity. At the same time, after adjusting the relationship between the proportion of Fe^3+^ and Fe^2+^ and the particle size of nanoparticles, it was found that there was a linear relationship between the two. The specific change pattern is y = (6.2 ± 0.4)x + (30.9 ± 8.2), where y is the particle size and x is the ratio of Fe^3+^ and Fe^2+^. Therefore, to some extent, we can regulate the ratio of Fe^3+^ and Fe^2+^ by adjusting the content of VC to obtain the required nanoparticle size. This all demonstrates the controllability of VC content on product particle size. The color of the products changed sequentially from brown to grey to black (Figure 1c), and all exhibited magnetically responsive photonic crystal diffraction effects, displaying bright colors under a magnet (Figure 1d). Thus, it was further verified that Fe^2+^ is an indispensable component for the synthesis of monodisperse Fe_3_O_4_. However, when the VC addition was 50 mg, the synthesized product neither settled nor showed optical diffraction under the magnet, probably because the particle size was too small and prone to agglomeration, resulting in slightly poor monodispersity.

### 3.2. Analysis of Crystal Size and Chemical Composition of Products

**Figure 2:**
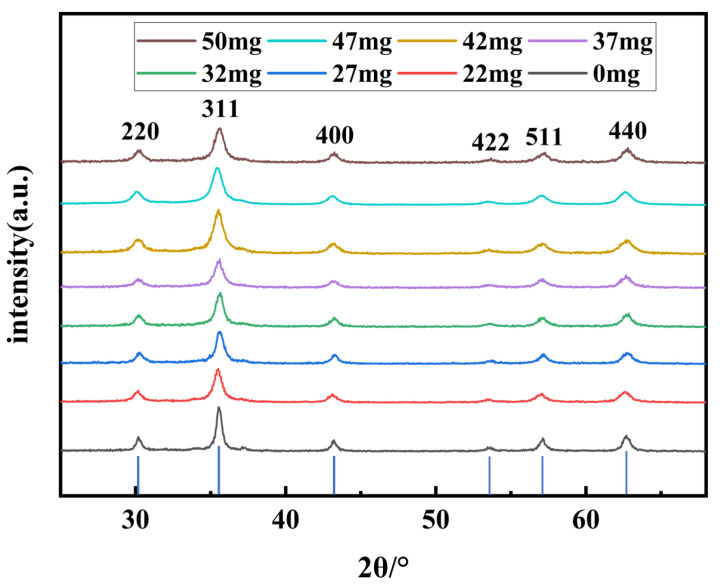
XRD patterns of synthesized products with varying VC addition amounts.

In order to analyze the effect of varying VC additions on the crystal size of the products as well as to study the chemical composition of different synthesized products, the groups of samples were characterized and tested. The XRD results are shown in Figure 2; the positions of the primary diffraction peaks in the synthesized products correspond to the peaks in the standard diffraction card JCPDS no.89-0688 of Fe_3_O_4_. With the gradual increase of VC addition, the diffraction peaks represented by the 311-crystal plane gradually become broader, indicating that the primary particle size of the synthesized Fe_3_O_4_ particles gradually decreases. Based on the Debye–Scherrer formula: D_hkl_ = kλ/βCos(θ), the Scherrer constant k is usually 0.89, λ is the wavelength of the incident X-ray. Cu target K is selected during testing α Ray, its wavelength λ is 0.15418 nm. β and θ are the half width of the diffraction peak and the Bragg diffraction angle, respectively. Meanwhile, the refraction and reflection of incident light on the crystal can cause a phase change in the scattered waves, resulting in coherent scattering regions. The primary crystal sizes of each Fe_3_O_4_ nanoparticle were calculated to be 39 nm, 23 nm, 21 nm, 17 nm, 16 nm, 13 nm, 11 nm, and 10 nm, respectively, when the addition of VC to the system varied from 0 to 50 mg. The primary crystal sizes of the nanoparticles were all in accordance with the critical size of the superparamagnetic nano Fe_3_O_4_ (30 nm) when VC was added to the system [45]. However, the Fe_3_O_4_ synthesized without VC in the system has a larger primary crystal size due to the presence of impurities, exceeding its critical size and with no superparamagnetism.

**Figure 3:**
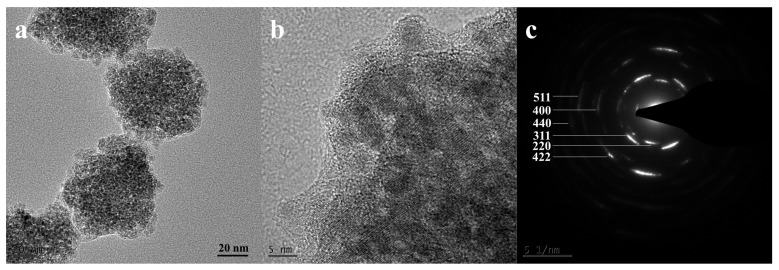
Product with a VC addition of 37 mg: (**a**) SEM image; (**b**) partial enlarged image; (**c**) SAED diagram.

The morphology and selected area electron diffraction of the product synthesized with a VC addition of 37 mg were studied by TEM. As shown in Figure 3a, the product exhibits an approximate spherical shape, with the average size of a single sphere being 120 nm. Figure 3b is a locally enlarged image, which shows that the single sphere is formed by the aggregation of countless small primary grains. Figure 3c is the SAED diagram of the product, and the electron diffraction pattern shows multiple concentric rings, indicating that the product has a polycrystalline structure. At the same time, the diffraction ring distribution corresponds to the crystalline type represented by the diffraction peak positions displayed in the above XRD, further suggesting that it is consistent with the Fe_3_O_4_ crystal structure.

**Figure 4:**
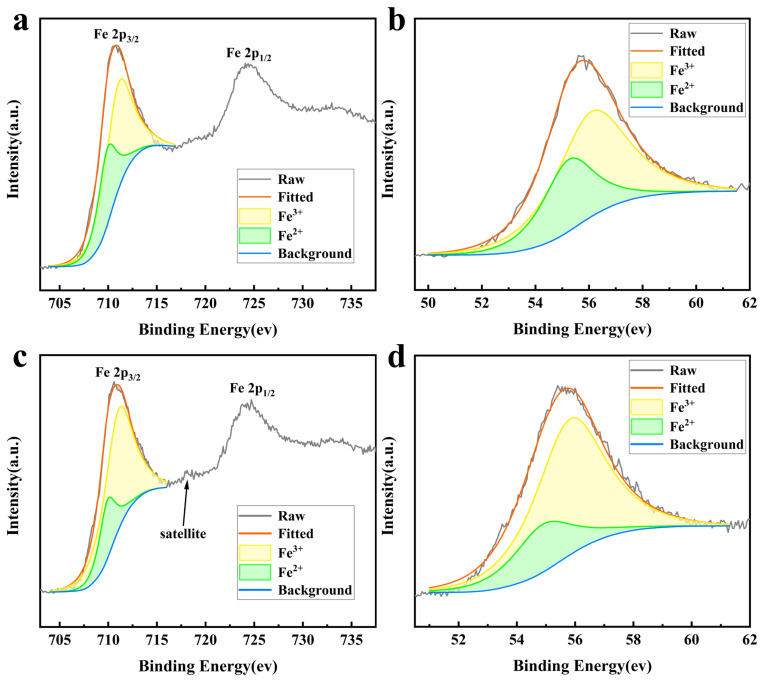
XPS spectra of different products: (**a**,**b**) show the Fe 2p and Fe 3p spectra of the synthesized products with 37 mg of VC added, respectively; (**c**,**d**) show the Fe 2p and Fe 3p spectra of the synthesized products without VC, respectively.

Further research was conducted on the chemical composition of the synthesized products under different conditions. The products synthesized from the precursor without VC and with 37 mg of VC were subjected to XPS tests, and the corresponding Fe 2p and Fe 3p split peaks were fitted as shown in Figure 4. The XPS of the product synthesized by adding VC to the precursor is shown in Figure 4a,b, with peaks appearing at positions 710.8 ev and 724.3 ev, corresponding to Fe 2p3/2 and Fe 3p1/2, respectively. A split-peak fit was carried out for Fe 2p3/2 and the ratio of the areas of the convolution peaks represented by them was 100,378.4 and 50,519.09, respectively and calculated to be Fe^3+^: Fe^2+^ = 2:1, which is consistent with the stoichiometric ratio between Fe^3+^ and Fe^2+^ in Fe_3_O_4_. Meanwhile, the peak at position 55.7 ev represents Fe 3p, and after fitting the peaks and calculating the area of 27,962.4 and 13,955.2, respectively, it was concluded that the ratio of Fe^3+^ and Fe^2+^ is also 2:1. Therefore, it indicates that the synthesized product with the addition of VC is a pure Fe_3_O_4_ nanoparticle without any other iron-containing impurities. Figure 4c,d shows the XPS spectra of the products synthesized without the addition of VC in the precursor, in which the peaks of Fe 2p and Fe 3p appeared at almost the same positions. But the ratio of the areas of their peaks represented by Fe^3+^ and Fe^2+^ was found to be about 3.5:1 after the fitting calculations with areas of 68,946.71 and 19,467.08, 30,523.6 and 8765.4, respectively, which did not correspond to the stoichiometric ratio of Fe^3+^ to Fe^2+^ in Fe_3_O_4_, indicating that there were other Fe-containing impurities in the products. Meanwhile, there was a small peak at about 718.2 ev in the Fe 2p spectrum, which was a satellite peak representing γ-Fe_2_O_3_ [46], and it could be further determined that there were γ-Fe_2_O_3_ impurities in this product.

Based on the above particle size, XRD, TEM, and XPS test results, it could be determined that when a certain amount of VC is added to the precursor, the synthesized product was a pure monodisperse nano Fe_3_O_4_, which inhibited the generation of γ-Fe_2_O_3_ impurities and had a diffraction effect for the MRPCs. Second, we could regulate the size of the synthesized nano Fe_3_O_4_ by the amount of VC, and the size of the nano Fe_3_O_4_ became smaller as the content of VC increased.

### 3.3. Magnetic Property Analysis of Nano Fe_3_O_4_

**Figure 5:**
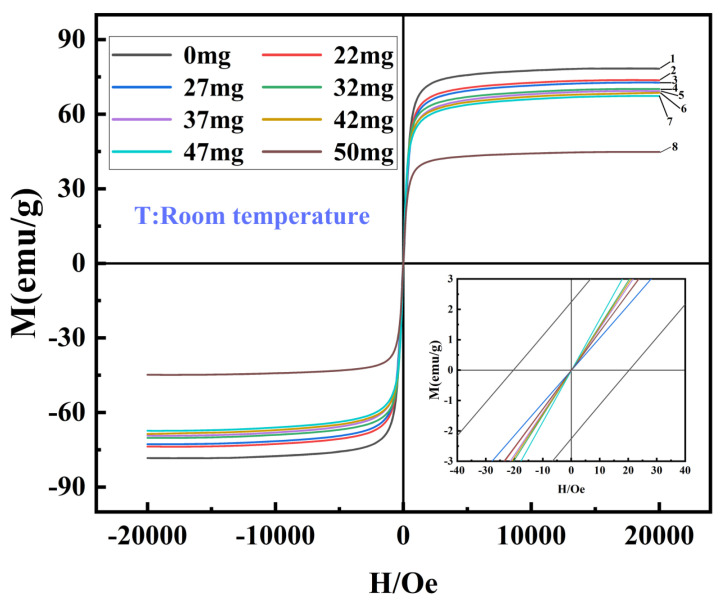
Magnetization curves of nano Fe_3_O_4_ synthesized with varying VC contents at room temperature.

The magnetic properties of nano Fe_3_O_4_ were further tested at room temperature, and the magnetic regression curves of nano Fe_3_O_4_ synthesized with different contents of VC are shown in Figure 5. The saturation magnetization intensity of nano Fe_3_O_4_ decreased from 75 emu/g to 45 emu/g when the addition of VC was increased from 22 mg to 50 mg. This was because the primary particle size of nano Fe_3_O_4_ decreases with the increase of VC content, and more atoms on the surface of the particles with a smaller particle size tend to lead to their disordered spin thus affecting the magnetic properties [47]. Moreover, the magnetization curves of these products all pass through the origin (as shown in the bottom right corner of the figure), without remanent magnetization and coercivity, and exhibit superparamagnetism at room temperature. The magnetization curve of the product synthesized without VC did not pass through the origin (shown in the lower right corner of the figure), and there was obvious remanent magnetism and coercivity, with values of 2.3 emu/g and 21 Oe, respectively, exhibiting some soft magnetism at room temperature. These are consistent with the results of the previous crystal size test.

### 3.4. Analysis of Nucleation Mechanism of Nano Fe_3_O_4_ Based on Reduction Method

**Figure 6:**
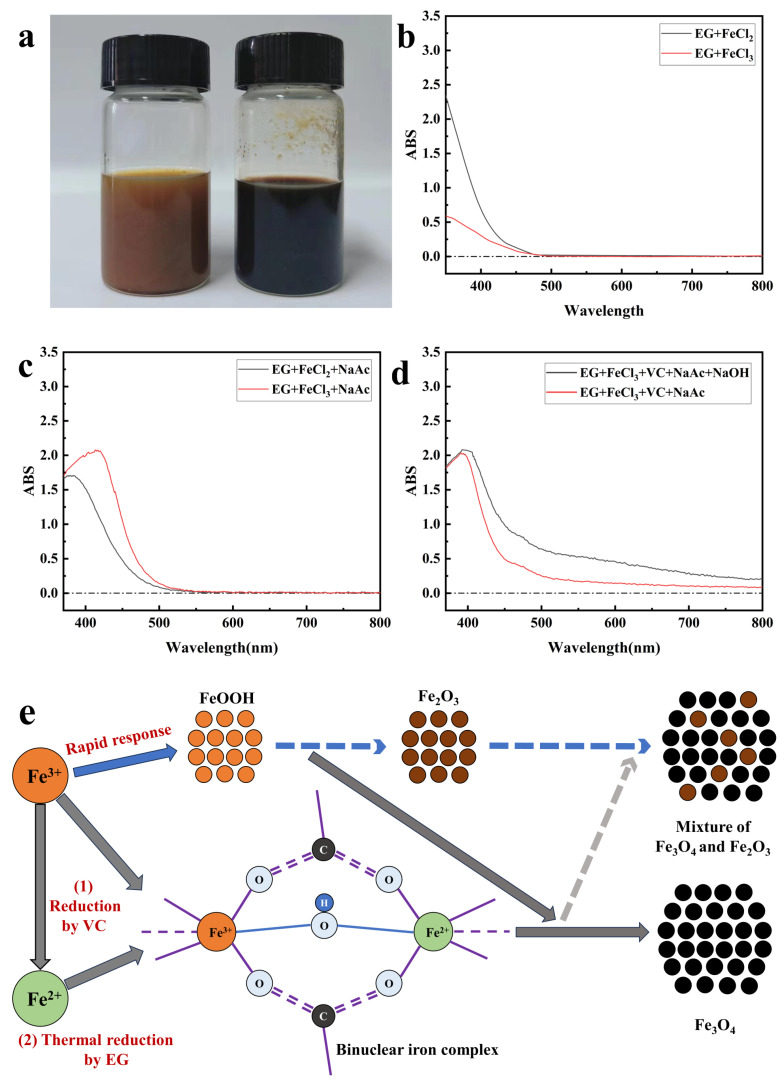
(**a**) Photo of the precursor (without adding VC on the left, with VC added on the right); (**b**,**c**) UV absorption spectrum when only Fe^3+^ or Fe^2+^ was present in the precursor; (**d**) UV absorption spectrum of Fe^3+^ and Fe^2+^ coexisted in the precursor; (**e**) schematic diagram of the synthesis mechanism of nano Fe_3_O_4_ based on reduction method.

The above tests indicate that the addition of VC to the precursor can synthesize superparamagnetic nano Fe_3_O_4_ with controllable particle size and monodispersity. In order to further investigate the nucleation mechanism of nano Fe_3_O_4_ synthesized by the addition of VC as well as the reason for the controllability of the particle size, the prepared precursor was characterized and tested. Photographs of the precursors obtained under different conditions are shown in Figure 6a, the left side without added VC presented a reddish brown color and the right side with added VC presented a black color. The UV absorption peaks of the precursors with different compositions were further tested as shown in Figure 6b–d. Only when Fe^3+^ and Fe^2+^ coexisted in the system, could the absorption bands formed cover the whole visible spectrum. This suggested that the addition of VC reduced part of Fe^3+^ to Fe^2+^, which resulted in characteristic absorption peaks in the entire visible range due to charge transfer when ions of different valence states coexist [48,49]. Meanwhile, when sodium hydroxide is added to the system, the broad-spectrum absorption peak had a higher absorbance, indicating that sodium hydroxide facilitated the charge transfer between Fe^3+^ and Fe^2+^. This broad-spectrum absorption was consistent with the phenomenon produced by mixed valence binuclear complexes [50,51,52], which was the formation of binuclear iron complexes in the system. The specific synthesis mechanism is shown in Figure 6e, where Fe^3+^ was eventually converted to FeOOH in an alkaline environment, and if VC was not added to the system at this time, then inevitably FeOOH would be rapidly generated to Fe_2_O_3_ impurities mixed in the final product; if VC was added to the system, a binuclear iron complex would be formed, and then FeOOH would be immediately combined with the complex to further generate pure Fe_3_O_4_ at high temperature and avoid the generation of Fe_2_O_3_ impurities. This was consistent with the nucleation theory of binuclear iron complexes proposed by our group [44]. Therefore, as the addition amount of VC increased, more nucleating agents were formed for the binuclear iron complex, and the crystal size of the nanoparticles became smaller, resulting in a smaller final particle size of Fe_3_O_4_ nanoparticles.

### 3.5. Stability Analysis of Fe_3_O_4_ Colloidal Clusters

**Figure 7:**
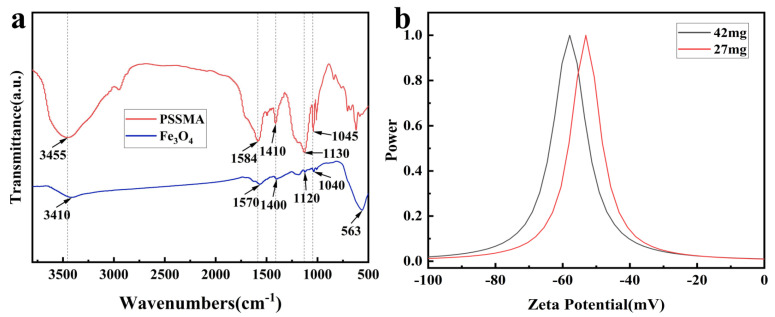
(**a**) Infrared spectra of Fe_3_O_4_ and PSSMA; (**b**) potential diagram of Fe_3_O_4_ synthesized with varying VC addition amounts.

When the amount of VC was increased from 22 mg to 50 mg, the synthesized Fe_3_O_4_ nanoparticles could be stably dispersed in water for two months, and although a small amount of delamination occurred later on, the nanoparticles could be stably dispersed in water again after sonication. When no VC was added, the synthesized Fe_3_O_4_ nanoparticles settled in a few days and were less stable. In order to investigate the stable dispersion of Fe_3_O_4_ colloidal clusters in water, infrared tests were first carried out on Fe_3_O_4_ synthesized with a VC addition of 37 mg with PSSMA, and the results are shown in Figure 7a. From the figure, it can be seen that the peak at 563 cm^−1^ is attributed to the Fe-O telescopic vibration peak in Fe_3_O_4_. The peaks at 1040 cm^−1^ and 1120 cm^−1^ belong to the symmetric and antisymmetric telescopic vibration peaks of sulfonic acid groups on the surface of synthesized Fe_3_O_4_, respectively. While the peaks at 1400 cm^−1^ and 1570 cm^−1^ were attributed to the symmetric and antisymmetric telescoping vibrational peaks of carboxylic acid groups on the synthesized Fe_3_O_4_ surface, respectively. The two peaks on Fe_3_O_4_ exhibited a blue shift compared to those in PSSMA, indicating that the two negatively charged groups were successfully attached to the surface of Fe_3_O_4_ through coordination by giving it electrostatic repulsion. The zeta potential of the synthesized Fe_3_O_4_ nanoparticles in water was then tested as shown in Figure 7b. The results showed that the potentials of the two kinds of Fe_3_O_4_ nanoparticles in normal water were −53 mV and −58 mV, respectively, which were much more than the minimum potential standard (±25 mV) for stable dispersion of colloids in solvent relying on electrostatic forces; this indicated that the Fe_3_O_4_ nano colloids were dispersed in water by electrostatic repulsive force and had strong stability. Therefore, the nano Fe_3_O_4_ synthesized in this chapter based on the reduction method had good monodispersity, superparamagnetism, and high surface charge stability at the same time, which enabled it to be instantaneously assembled into a photonic crystal dot matrix structure under a magnetic field.

### 3.6. Analysis of Diffraction Performance of Fe_3_O_4_ MRPCs

**Figure 8:**
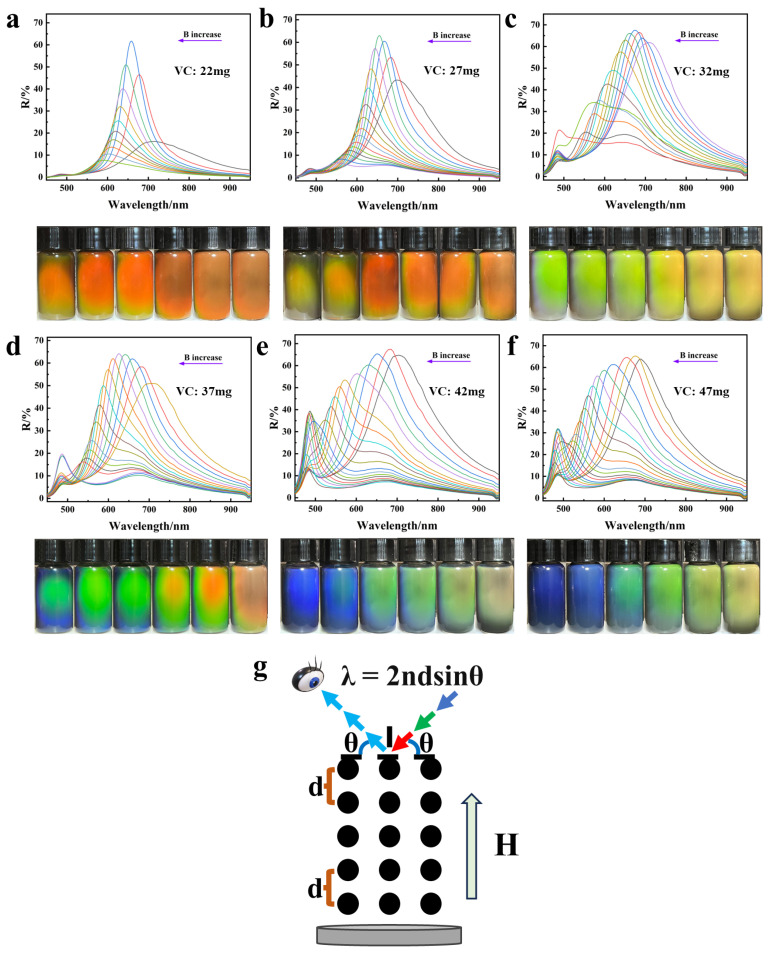
(**a**–**f**), respectively, show the reflection spectra and optical photos of the nano Fe_3_O_4_ aqueous dispersion with the particle size gradually decreasing under a magnetic field of 100–600 Gs; (**g**) schematic diagram of diffraction principle.

**Figure 9:**
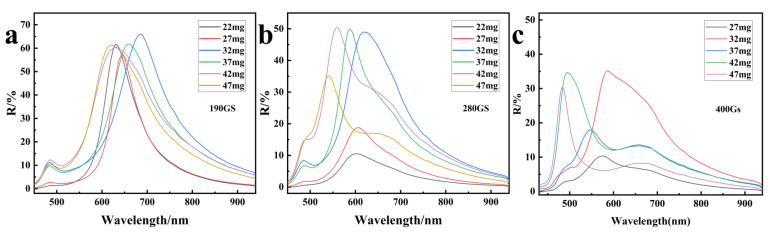
Reflection spectra of MRPCs of Fe_3_O_4_ with different particle sizes under the same magnetic field: (**a**) 190 Gs; (**b**) 280 Gs; (**c**) 400 Gs.

In order to investigate the tuning effect of the synthesized Fe_3_O_4_ MRPCs on the diffracted light, the reflectance spectra and optical photographs of the synthesized Fe_3_O_4_ colloidal nanoclusters with varying VC additions were tested in the range of magnetic field strengths from 100–600 Gs, as shown in Figure 8a–f. The diffraction wavelengths of each sample were gradually blue-shifted with the gradual increase of the magnetic field strength. The principle of diffraction is shown in Figure 8g; with the gradual increase of the magnetic field, the distance between the particles decreased accordingly, and the diffracted wavelengths were gradually blue-shifted according to the Bragg formula. Figure 9 shows the diffraction spectra of nano Fe_3_O_4_ with different particle sizes under the same magnetic field. The diffraction spectra were first red-shifted and then blue-shifted with the gradual decrease of the particle size of nano Fe_3_O_4_ under the same magnetic field. Moreover, the larger the strength of the magnetic field, the wider was the range of the wavelength shift and the overall gradual blueshift—the range of diffraction wavelength shift was 60 nm, 80 nm, and 103 nm in that order. Generally speaking, as the particle size decreased, the distance between particles also decreased relatively under the same magnetic field. But at the same time, the magnetic attraction generated by the magnetization of the particles also became weaker, which led to an increase in the distance between particles under the condition of constant electrostatic repulsive force. In addition, the surface electrostatic repulsion of nano Fe_3_O_4_ synthesized with varying VC contents was also different. Therefore, the combined effect of particle size, magnetization attraction, and surface electrostatic repulsion caused the diffraction wavelength to be red-shifted and then blue-shifted with the gradual decrease of nano Fe_3_O_4_ particle size under the same magnetic field. In addition, with the strengthening of magnetic field, the range of changes brought about by these three factors were larger, so the range of the diffraction wavelength shift became relatively wider.

### 3.7. Modification of Nano Fe_3_O_4_ and Analysis of MRPCs Diffraction Performance in Organic Solvents

**Figure 10:**
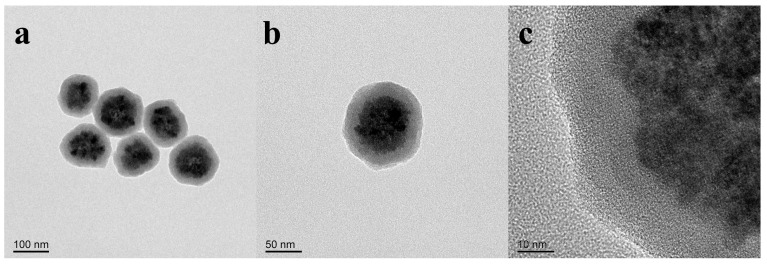
TEM images of modified nano Fe_3_O_4_ at different magnifications (**a**–**c**).

The long-term stability of nano Fe_3_O_4_ synthesized by the solvothermal method is insufficient and basically dispersed only in aqueous solution, which limits its further application. In order to make the nano Fe_3_O_4_ stably dispersed in non-aqueous solvents and assembled into photonic crystal structures for specific application performance, the synthesized nano Fe_3_O_4_ was modified by encapsulating silicon under the effect of PVP. The results of the TEM test on the nano Fe_3_O_4_ synthesized by the addition of VC with a dosage of 42 mg after the modification are shown in Figure 10. As can be seen from the figure, the surface of nano Fe_3_O_4_ was successfully coated with a layer of SiO_2_ shell, forming a spherical structure of core-shell, and the thickness of the shell layer was about 25 nm. The overall single sphere showed that SiO_2_ was very uniformly coated on the surface of nano Fe_3_O_4_, and the overall diameter of the sphere was about 120 nm. Second, the interior of the structure was composed of spherical Fe_3_O_4_ particles aggregated with small particles, exhibiting a highly ordered structure as a whole.

**Figure 11:**
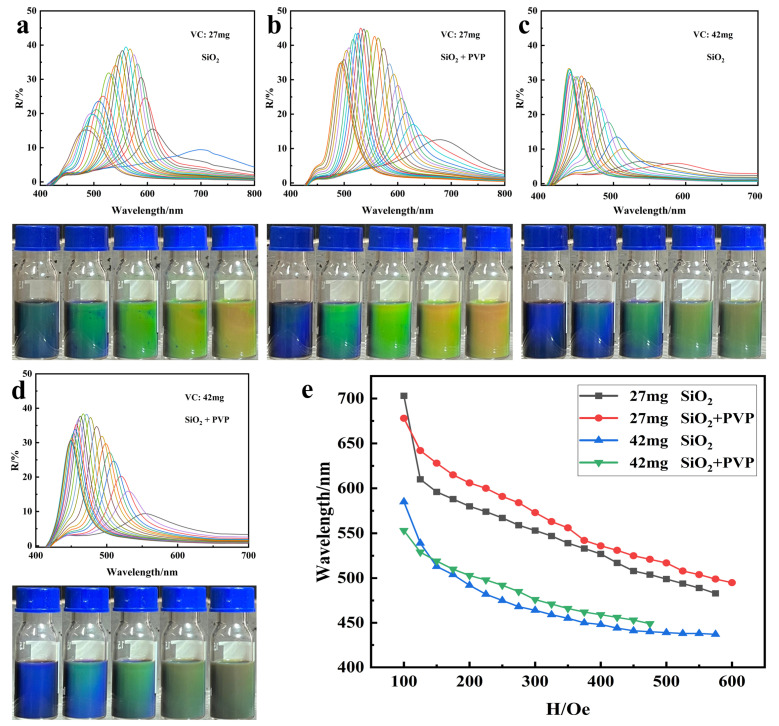
(**a**–**d**) Reflectance spectra and optical photos of Fe_3_O_4_ nanoparticles dispersed in ethanol with different modified conditions under the magnetic field of 100–600 Gs; (**e**) diffraction wavelength range of modified Fe_3_O_4_ nanoparticles dispersed in ethanol under magnetic fields.

The reflectance spectra and optical photographs of the modified nano Fe_3_O_4_ dispersed in ethanol under a magnetic field of 100–600 Gs are shown in Figure 11a–d. The diffraction wavelength of the same sample was gradually blue-shifted with the enhancement of the magnetic field strength, and the overall diffraction wavelength of the sample with smaller particle size was blue-shifted. The addition of PVP coated silicon resulted in stronger diffraction peaks in the reflectance spectra and the overall diffraction wavelength was relatively red-shifted compared with the normal silicon modification on nano Fe_3_O_4_. This is due to the fact that the PVP coating improved the overall homogeneity of the nano Fe_3_O_4_, presenting better monodispersity and resulting in stronger diffraction peaks. Second, the PVP along with SiO_2_ coating on the surface of nano Fe_3_O_4_ further enlarged its overall size, so that the distance between particles was relatively larger under the same magnetic field, which ultimately led to a relative red-shift of the overall diffraction wavelength, as shown in Figure 11e.

**Figure 12:**
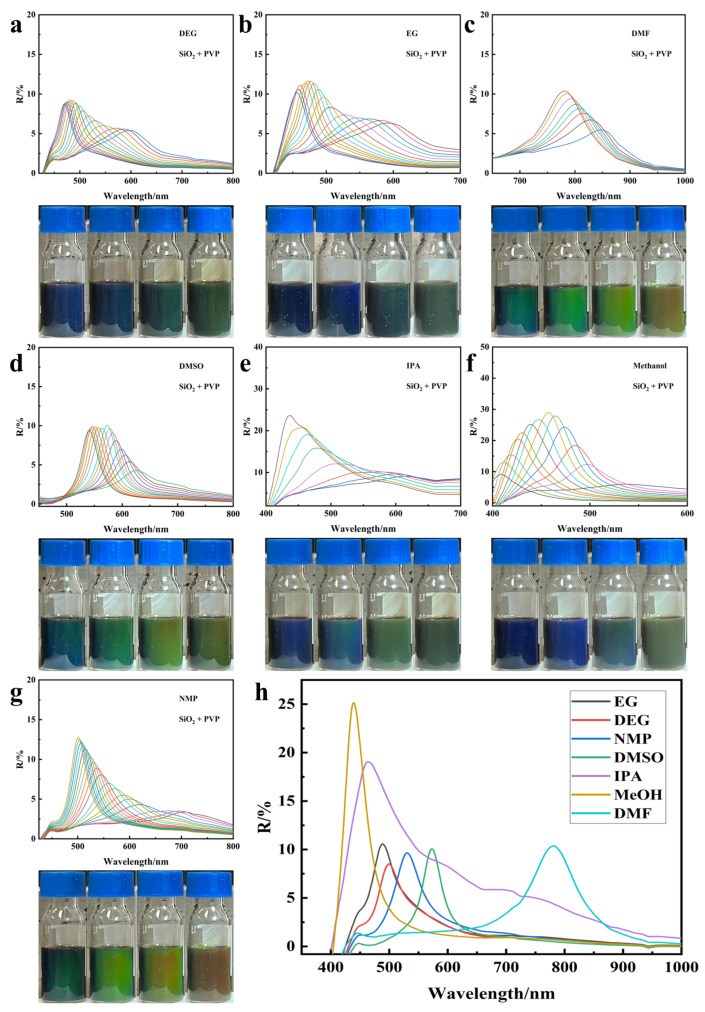
Reflection spectra and optical photos of nano Fe_3_O_4_ modified with PVP coated silicon dispersed in different solvents under the magnetic field of 100–600 Gs (**a**) DEG, (**b**) EG, (**c**) DMF, (**d**) DMSO, (**e**) IPA, (**f**) methanol, (**g**) NMP; (**h**) reflection spectra of modified nano Fe_3_O_4_ dispersed in different solvents under the same magnetic field of 400 Gs.

Nano Fe_3_O_4_ of the PVP coated silicon modified was sequentially dispersed in different organic solvents and was able to instantaneously assemble into photonic crystal structures under the magnetic field of 100–600 Gs. The reflectance spectra and optical photographs are shown in Figure 12a–g. The addition of PVP coated silicon modified nano Fe_3_O_4_ could be stably dispersed in a variety of organic solvents and be assembled into a photonic crystal structure to diffract wavelengths of different ranges. The diffracted wavelengths were blue-shifted with the increase of the magnetic field, which demonstrated its ability to tune diffraction light in multiple solvents. Second, the diffracted wavelengths in different solvents under the same magnetic field were also different as shown in Figure 12h, which was related to the different solvation ability of each solvent and the different conformation of PVP inside the solvent. The thickness of the solvation layer of each solvent was different; for example, the solvation layer of DMF, DMSO, etc. was thicker so that the nanoparticles were further separated resulting from the increase of their spacing, so the diffraction wavelength was in the red zone. In methanol, the solvent layer was also thicker, but the PVP on the surface of the particles in methanol presented an entangled conformation so that the thickness of the solvent layer was compressed, eventually leading to a smaller distance between the particles, so that the diffraction wavelength was in the blue region. Therefore, the nano Fe_3_O_4_ modified by PVP coated silica can be dispersed in a variety of organic solvents, and it is expected to be used in a variety of scenarios in the field of optical color development.

## 4. Conclusions

In this study, we designed and investigated a reduction-based method to synthesize controllable monodispersed superparamagnetic nano Fe_3_O_4_ for MRPCs. VC was used as a reducing agent to control the reduction of part of Fe^3+^ to Fe^2+^ in the system to form binuclear iron complexes, which resulted in the generation of superparamagnetic nano Fe_3_O_4_ with controllable particle size and polydispersity. It was shown that the synthesized nano Fe_3_O_4_ was poorly monodispersed and generated γ-Fe_2_O_3_ impurities when VC was not added to the system and could not be assembled into photonic crystals under a magnetic field. When VC was added, the synthesized Fe_3_O_4_ nanoparticles exhibited monodispersity and superparamagnetism, while the particle size and saturation magnetization intensity decreased gradually with the increase of VC. The particles could be assembled rapidly into photonic crystals under a magnetic field with the dot-matrix structure, displaying the tunability to various diffraction wavelengths. The monodispersity and stability of Fe_3_O_4_ nanoparticles were further improved by PVP coated silicon modification, and the particles could be stably assembled into photonic crystal dot matrix structures in various organic solvents under a magnetic field. This is expected to be applied in the field of optical color development in various scenarios.

## Figures and Tables

**Table 1:** Performance of synthesized products with different amounts of VC added to precursors.

		Precursor		Product		
No.	VC/mg	Fe^2+^ by Reduction/mmol	Fe^3+^/Fe^2+^	Particle Diameter/nm	PDI	Magnetochomc Effect
1	0	0	0:0	347.1 ± 4.3	0.227	No
2	22	0.25	32:1	223.4 ± 3.6	0.061	Yes
3	27	0.31	26:1	193.9 ± 3.8	0.059	Yes
4	32	0.36	22:1	176.9 ± 2.4	0.045	Yes
5	37	0.42	19:1	151.9 ± 2.8	0.043	Yes
6	42	0.48	17:1	136.6 ± 3.2	0.048	Yes
7	47	0.53	15:1	122.2 ± 2.0	0.033	Yes
8	50	0.57	14:1	111.3 ± 2.4	0.179	No

## Data Availability

Data are contained within the article.
